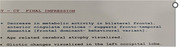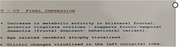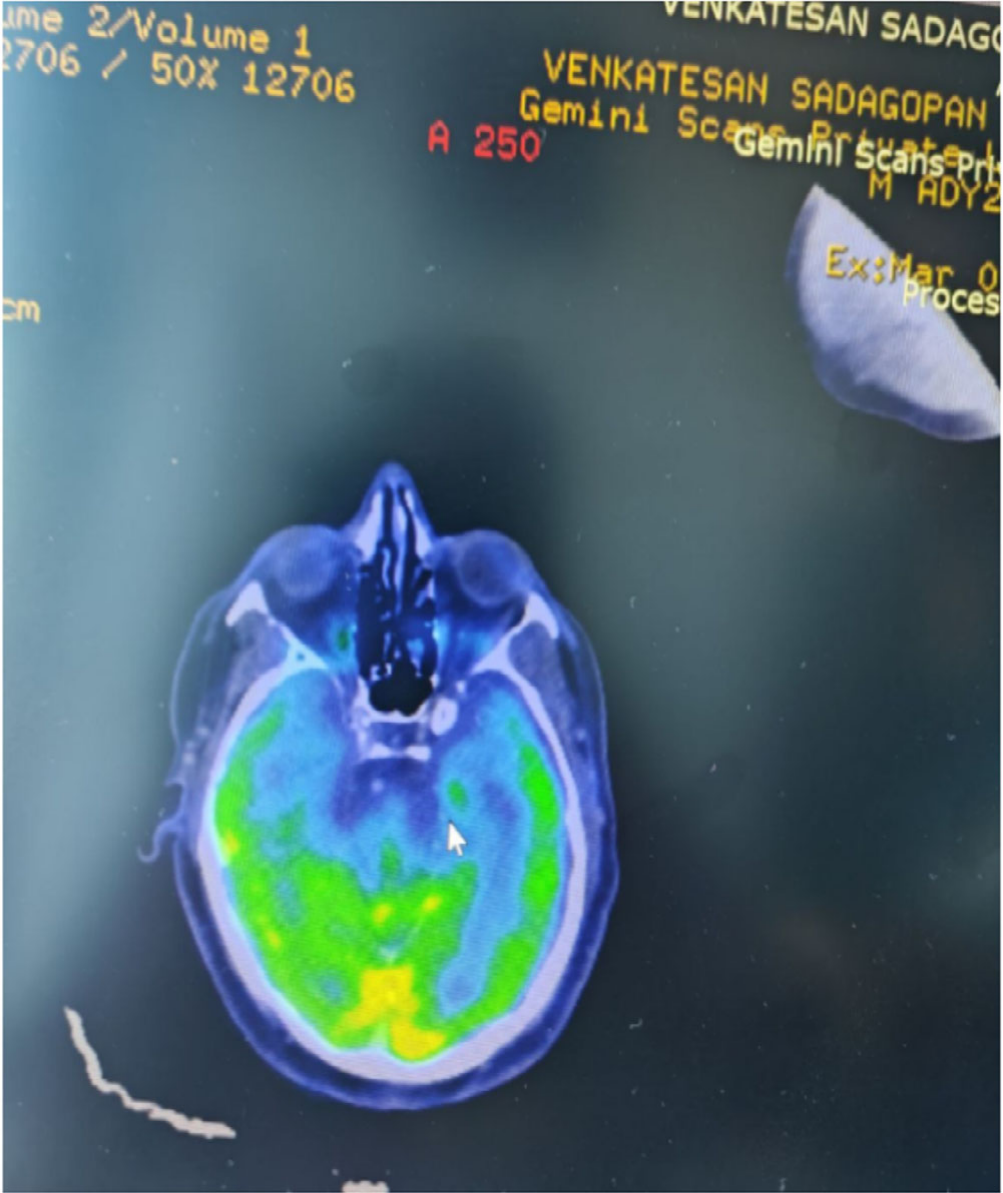# Beyond the tremors, when Parkinsonism masks FTLD

**DOI:** 10.1002/alz70857_103567

**Published:** 2025-12-24

**Authors:** Swathi T, Subramaniyan K

**Affiliations:** ^1^ Kauvery hospital, Chennai, Tamil nadu, India; ^2^ Kauvery hospital, Chennai, Tamilnadu, India

## Abstract

**Background:**

FTLD presents with executive function impairment along with memory impairment semantic memory. Herein we present one such case who presented with Parkinsonism features, however PET CT has the trust

**Method:**

A 60 year male presented with complaints of slowness of walking, slowness of activity, with staring look over 1 years with further progression to repetitive spech, apathy, impatient of activity of daily living with insomnia. On examination patient had extrapyramidal features with perseveration, echolalia, palilalia.

A possibility of treatable condition paraneoplastic syndrome / neurodegenerative Parkinson plus was considered.

**Result:**

Autoimmune and paraneoplastic profile negative. PET CT done which was digestive of FTLD.

**Conclusion:**

HEREIN we present a case of FTLD which was marked by Parkinson feature.